# A Descriptive Analysis of HIV Prevalence, HIV Service Uptake, and HIV-Related Risk Behaviour among Patients Attending a Mental Health Clinic in Rural Malawi

**DOI:** 10.1371/journal.pone.0072171

**Published:** 2013-08-28

**Authors:** Kinke Lommerse, Robert C. Stewart, Queen Chilimba, Thomas van den Akker, Crick Lund

**Affiliations:** 1 Thyolo District Health Office, Ministry of Health, Thyolo, Malawi; 2 Department of Psychiatry, GGZinGeest, Amsterdam, The Netherlands; 3 Department of Mental Health, College of Medicine, University of Malawi, Blantyre, Malawi; 4 Centre for Public Mental Health, Department of Psychiatry and Mental Health, University of Cape Town, Cape Town, South Africa; Chancellor College, University of Malawi, Malawi

## Abstract

**Background:**

Human immunodeficiency virus (HIV) and mental illness are interlinked health problems; mental illness may pose a risk for contracting HIV and HIV-positive individuals are at higher risk of mental illness. However, in countries with high HIV prevalence, the main focus of HIV-related health programmes is usually on prevention and treatment of somatic complications of HIV, and mental illness is not given high priority. We examined HIV prevalence, uptake of HIV services, and HIV-related risk behaviour among people attending a mental health clinic in rural Malawi.

**Methodology:**

Semi-structured interviews were performed with patients capable to consent (94%), and with those accompanied by a capable caregiver who consented. HIV counselling and testing was offered to participants.

**Findings:**

Among 174 participants, we collected 162 HIV test results (91%). HIV prevalence was 14.8%. Women were three times as likely to be HIV-positive compared to men. Two-thirds of participants reported having been tested for HIV prior to this study. The uptake of HIV-services among HIV-positive patients was low: 35% did not use recommended prophylactic therapy and 44% of patients not receiving antiretroviral treatment (ART) had never been assessed for ART eligibility. The reported rate of sexual activity was 61%, and 9% of sexually active participants had multiple partners. Inconsistent condom use with stable (89%) and occasional (79%) sexual partners, and absence of knowledge of the HIV status of those partners (53%, 63%) indicate high levels of sexual risk behaviour.

**Conclusions:**

HIV-prevalence among persons attending the clinic, particularly men, was lower than among the general population in a population survey. The rate of HIV testing was high, but there was low uptake of preventive measures and ART. This illustrates that HIV-positive individuals with mental illness or epilepsy constitute a vulnerable population. HIV programmes should include those with neuropsychiatric illness.

## Introduction

Globally, the burden of disease attributable to mental, behavioural and neurological disorders is rising and calculated at 10.4% of all disability-adjusted life years [1]. More than three quarters of this burden occurs in low- and middle-income countries, where resources for mental health, including policy, financing, community structures and health staff, are often insufficient [2].

Resource limitations have become more apparent in the context of the HIV pandemic. The overwhelming demand for HIV care has drawn heavily upon available financial and human resources. In countries with a high HIV prevalence, the focus has been on the prevention and treatment of somatic complications, although people infected with HIV are also at a higher risk of developing mental illness [3]. Mental illness, in turn, can be a risk factor for contracting HIV infection [3]. Therefore, there is likely to be a considerable unmet need for HIV-care among people with mental illness, and for mental health care among HIV-infected persons [4].

Several studies from developed countries have reported that adults with severe mental illness have elevated rates of HIV-infection [5]–[8]. Despite the high HIV burden in several low- and middle-income countries, little evidence is available about the relationship between mental illness and the risk to contract HIV in these areas. The few HIV-seroprevalence studies among people with mental illness in psychiatric hospitals found HIV prevalence rates corresponding to the prevalence in the general population [9]. Two studies from sub-Saharan Africa (Zimbabwe and South Africa) showed extremely high infection rates of 23.8% and 26.5% among the in-patient population of mental health hospitals, which corresponded with the high rates in the general population [4]; [10].

In high-income countries this increased HIV prevalence is associated with high-risk behaviour such as drug injection use and high-risk sexual behaviour [11]–[14]. In low-income countries with high HIV prevalence this association is less clear.

Councelling and testing for HIV is an important entry point for HIV care, especially since people with mental disorders may delay seeking health care and are less likely diagnosed with other health conditions [3]. In high income countries, HIV testing rates among people with severe mental illness between 17% and 47% in the year prior to study have been found [15]. Data from low-income countries are non-existent. Some previous reports have indicated that clinicians may refrain from testing patients with severe psychiatric symptoms, but other studies could not confirm this [15].

In Malawi, a country with one of the highest HIV prevalence rates in the world, there is a lack of documentation of HIV prevalence among people suffering from neuropsychiatric disorders. Extrapolating data from other countries, a substantial part of the Malawian population is likely to suffer from neuropsychiatric disorders. As in many low- and middle-income countries, mental health services in Malawi are concerned with neuropsychiatric disorders: mental, behavioural, and neurological conditions, such as epilepsy.

The aim of this study was to systematically examine HIV prevalence, HIV service uptake and HIV-related risk behaviour among patients with neuropsychiatric disorders attending a mental health clinic in rural Malawi.

## Materials and Methods

### Study Design

This was a descriptive cross-sectional study of patients attending a mental health and epilepsy clinic.

### Study Period and Setting

The study was conducted from August 1st, 2009, to May 1st, 2010, in Thyolo District, a predominantly rural area in Southern Malawi, a very low-income country in sub-Saharan Africa. The district population was estimated at 617,000 in 2010 and had an HIV-prevalence rate of 21% in 2004 [16]. The publicly funded Thyolo District Hospital is the largest health facility and the most important secondary referral center in the area. At the extremely busy outpatient department, which is both a primary care clinic for the local catchment area and a referral facility for peripheral health centres, a weekly mental health clinic for psychiatric disorders and epilepsy is held. Here, an average of 75 clients are seen in one morning. Occasionally, in case patients are not able to attend the clinic themselves, their caregivers come and collect medication.

Inpatient mental health care is delivered at the district hospital wards, and irregular outreach clinics take place throughout the district. Most mental health care in the district is provided by nurses trained in basic mental health care. There is a central referral hospital for mental health, Zomba Mental Hospital, located one hundred kilometres away. Referral from Thyolo to Zomba occurs in case of severe psychiatric symptoms, and can be seen as an indicator for severity. Diagnoses are usually given by the psychiatric nurses and documented in the patient’s ‘health passport’. The number of diagnoses applied is limited. However, in absence of specialist care, this basic list is practical in the local setting. Medication from the essential health package for common neuropsychiatric conditions is available.

In Thyolo District, ART became available in 2003 and district-wide coverage was achieved in 2007 [17]. Malawi has been making use of a simple and standardised first-line ART regimen. HIV testing and counselling (HTC) services are available at community, health centre and hospital levels [18]. Counselling is done by minimally trained counsellors. HTC is voluntary and not routinely offered to patients, except at the antenatal clinic. Until recently, national HTC guidelines provided only minimal advice about the testing of people with mental illness [19].

### Study Participants

A study sample of 156 was calculated to assess a difference in the HIV-prevalence among the clinic population compared to the general population, with a 95% confidence interval and 5% precision. The general population prevalence in Thyolo District of 21% was taken from the District Health Survey (DHS) [16]. HIV prevalence is monitored through antenatal clinic sentinel surveillance.

Patients attending the clinic who were age eighteen years or above were included. Excluded were: (A) those under the acute influence of alcohol or drugs, (B) those with an acute disturbance of the mental state, (C) those that suffered from a physical condition for which they required admission or (D) those with a severely impaired mental capacity without an accompanying caregiver.

Participants were randomly included. Persons in a strictly kept queue were assigned to either the room for routine check-up or the study room, depending on which was available for the next consultation. This procedure continued until all persons in the queue had been seen in one of the rooms. The consultation started with an interview in which the primary investigator (KL), who is a general physician with mental health experience, judged eligibility and capacity to provide informed consent. When a patient lacked capacity to provide consent, a committed caregiver was asked to give consent on behalf of the patient. The purpose of the research was explained, and the patient or caregiver was invited to read the information sheet and consent form in Chichewa or this was read out to illiterate patients. Written consent was obtained from the patient or caregiver or their thumbprint was used.

### Data Collection and Analysis

After obtaining informed consent, semi-structured interviews were performed. Information collected and categorised included: neuropsychiatric diagnosis and treatment (taken from the participant’s ‘health passport’), socio-demographic factors (age, sex, marital status, number of children, highest school level, income generation, type of job, income per month, availability of household goods), variables for the neuropsychiatric condition (years since onset of disorder, ever admitted in referral psychiatric hospital, in time for drug refill previous five visits, number of seizures in the previous month), general health perception and problems (health problems other than HIV, history of sexual transmitted infection (STI) treatment, history of tuberculosis (TB) treatment), HIV/AIDS services utilization (history of HTC with or without documentation, HIV test result before inclusion into the study, use of co-trimoxazole prophylaxis, use of ARVs) and HIV-related risk behaviour. With regard to risk behaviour we asessed alcohol use, binge drinking, cigarette and drug use in the previous month. In addition, we interviewed participants about sexual risk behaviour in the previous year. Questions included the type of relationship (married, engaged), number of other sexual partners, condom use with all sexual partners (always, sometimes, never) and knowledge about HIV status of sexual partners. ‘Sex’ was not specified to the participants. When participants lacked capacity to consent and were accompanied by a caregiver, sexual behaviour was discussed without the caregiver’s presence.

HCT was offered. Results were de-identified and documented in a patient record. HTC was done in a dedicated room that ensured privacy. Counsellors received an additional one-day training about HIV counselling for mental health patients from the first author (KL) and an experienced psychiatrist (RS). Counselling and testing were provided according the National HIV/AIDS counselling and testing Guidelines [19].

We accepted three types of HIV test results: (1) a documented test result obtained in a recognized HTC clinic within three months prior to inclusion, and (2) a previously properly documented *positive* HIV-test result (3) a test result obtained after referral for HTC as part of the study. For patients who refused referral for HTC we documented the reason given.

For HIV testing, the local HIV-testing algorithm and assays were used. The district used an algorithm of three simple rapid HIV assays, which is based on the use of Determine™ HIV-1/2 assay for screening, followed by confirmatory testing of reactive samples by Uni-Gold™ HIV-1/2, with SD Biolin as the tiebreaker for discordant results.

All relevant data were entered in a Microsoft Excel database. Missing data were treated as missing in the analysis. The data were analyzed using the statistical programs: Openepi, Version 2.3 [20] and EpiCalc 2000, Version 1.02. The Chi squared (Fisher exact) test was used to examine if there were differences between patients with epilepsy and patients with psychiatric disorders in terms of their socio-demographic characteristics and mental health characteristics. The significance level was set at 5%.

Two-by-two tables were used to evaluate the association between a possible risk factor and HIV infection. Odds ratios and their 95% confidence limits were calculated with Fisher Exact method.

### Ethics Statement

The study was performed according to Malawian national guidelines. The Thyolo District Health Office of the Ministry of Health and the Department of Mental Health of the College of Medicine in Blantyre took part in the study design. We received ethical approval from the College of Medicine Research Ethics Committee in Malawi and the the Human Research Ethics Committee of the Faculty of Health Sciences of the University of Cape Town was informed about the study.

## Results

185 patients were assessed for eligibility. Of this group, six patients were found to be under the age limit, two had an acute disturbance of their mental state and three had severely impaired mental capacity without an accompanying caregiver. All of the remaining 174 patients or their caregiver consented to participating in the study.

### Diagnostic Profile

More than half of the participants had epilepsy (n = 99, 56.9%). One person in this group was also diagnosed with a developmental disorder. Almost half of the patients with epilepsy (48%) reported no convulsions in the previous month, and 47% reported between one and four convulsions.

Of 75 participants with a psychiatric diagnosis (43.1%), 56 had schizophrenia spectrum disorders (74.7%). Within this group, one patient had an additional diagnosis of alcohol misuse and one of drug misuse (cannabis). Other diagnoses (n = 20) included affective disorder (n = 7, 9.3%), and organic and acute psychosis (n = 8, 10.7%).

Most participants (n = 163, 93.7%) were assessed to have the capacity to provide informed consent. Patients who themselves lacked the capacity to consent and who needed caregivers to provide consent on their behalf were more often found in the group with psychiatric disorders than in the group with epilepsy (12% vs. 2%) (p = 0.018). Of these patients (n = 11) only one patient was not able to discuss sexual behaviour. In the group of participants with a psychiatric diagnosis, 39 (52%) had had a previous admission to the central psychiatric referral hospital. No participants with a diagnosis of epilepsy had been referred to central level. Regarding adherence to medication, most of the participants (n = 143, 82%) had not missed any of the five previous appointments for drug refill.

### Sociodemographic Profile

Sociodemographic characteristics are shown in table 1. Ages ranged from 18 to 85 years (mean age = 33.2 (SD 12.6)). Socio-demographic characteristics of the group of patients with epilepsy and with a psychiatric disorder were generally comparable. Two significant differences between both groups were found. Patients with psychiatric disorders were more likely to have an educational level of standard 5 or higher (n = 52 (69%)) compared to patients with epilepsy (n = 38 (38%)) (p = 0.0001), and had a significantly better income.

**Table 1:** Socio-demographic variables of patients with epilepsy and psychiatric disorders.

Variables	All N = 174	Patients with epilepsy N = 99		Patients with psychiatric disorders N = 75		Chi squared test(two-sided p-value)
	n (%)					
**Sex**						
Male	93 (53)	50 (51)			43 (57)	p = 0.46
Female	81 (47)	49 (49)			32 (43)	p = 0.46
**Age (years)**						
18–19	11 (6)	6 (6)			5 (7)	p = 0.88
20–24	36 (21)	21 (21)			15 (20)	p = 0.99
25–29	34 (20)	20 (20)			14 (19)	p = 0.95
30–34	28 (16)	13 (13)			15 (20)	p = 0.31
35–39	22 (13)	17 (17)			5 (7)	p = 0.06
40–44	12 (7)	7 (7)			5 (7)	p = 0.84
45–49	10 (6)	5 (5)			5 (7)	p = 0.90
≥50	21 (12)	10 (10)			11 (15)	p = 0.49
**Marital status**						
Single	42 (24)	24 (24)			18 (24)	p = 0.89
Married	85 (49)	47(47)			38 (51)	p = 0.79
Separated/divorced	43 (25)	26 (26)			17 (23)	p = 0.71
Widowed	4 (2)	2 (2)			2 (3)	p = 0.81
**Number of children**						
0	47 (27)	25 (25)			22 (29)	p = 0.66
1–2	58 (33)	38 (38)			20 (27)	p = 0.14
3–4	44 (25)	26 (26)			18 (24)	p = 0.87
5+	25 (14)	10 (10)			15 (20)	p = 0.10
**Education level**						
None	28 (16)	21 (21)			7 (9)	p = 0.06
Primary 1–4	49 (28)	33 (33)			16 (22)	p = 0.11
Primary 5–8	63 (36)	21 (21)			35 (47)	p = 0.0007
Secondary or more	34 (20)	17 (17)			17 (23)	p = 0.47
**Income generation**						
By participant	111 (63)	63 (64)			48 (64)	p = 0.91
**Type of work**						
Tea estate	25/111* (23)	18/63* (29)			7/48* (15)	p = 0.13
Farming	41/111 (37)	22/63 (35)			19/48 (40)	p = 0.76
Small business	22/111 (20)	12/63 (19)			10/48 (21)	p = 0.99
Other	23/111 (21)	11/63 (17)			12/48 (25)	p = 0.46
**Monthly income** **(in MKW** ** **)**						
<2000	20/111* (18)	17/63* (27)			3/48* (6)	p = 0.01
2000–3999	52/111 (47)	31/63 (49)			21/48 (44)	p = 0.70
4000–5999	15/111 (14)	6/63 (10)			9/48 (19)	p = 0.26
≥6000	19/111 (17)	6/63 (10)			13/48 (27)	p = 0.03
Not known	5/111 (5)	3/63 (5)			2/48 (4)	p = 0.75
**Household goods**						
Electricity	15 (9)	8 (8)			7 (9)	p = 0.98
Radio	94 (54)	50 (51)			44 (59)	p = 0.36
Cell phone	29 (17)	12 (12)			17 (23)	p = 0.10

* denominator is total inclusions in subgroup.

** MKW = Malawian Kwacha. MKW1000 =  US$6.

### HIV Service uptake and Pre-study Prevalence

Of participants, 110 (63.2%) reported to have been tested for HIV prior to the study, and 65 (37.3%) had documented evidence of a test result. Of those with documented evidence 17 (26%) had tested HIV-positive. Based on this, the pre-study recorded HIV prevalence was calculated at 9.8%.

Of the 17 individuals who tested positive prior to the study, six (35%) did not use co-trimoxazole prophylaxis according to national guidelines. Nine (53%) did not receive ART: five (56%) had their CD4 count assessed in the previous year and were found not eligible for ART, four (44%) had never been staged according to WHO staging criteria.

Patients with a psychiatric disorder and a history of severe symptoms (indicated by referral to the central mental hospital) showed a trend towards being more likely to have been tested for HIV compared to patients without referral (OR 2.3; CI = 0.96–5.91). There were no statistical differences between the patients with epilepsy and psychiatric disorders for uptake of HIV services and the pre-study prevalence of HIV.

### HIV Prevalence

Among 174 participants, 162 test results were collected. From twelve participants (6.9%) an HIV test result was not obtained: five did not return after going for HTC and seven refused testing. Reasons for refusal were: ‘no interest’, ‘want to discuss with partner or caregiver’, ‘presupposed knowledge about a negative HIV-status’ or, ‘too old to be tested’.

Of the 162, 24 were found to be HIV positive (14.8%; 95% CI = 9,73–21.24), 13 (54%) of whom were in the 25–34 age group. Women were three times more likely to be infected with HIV compared to men (OR 3.05; CI = 1.21–8.33; p = 0.027). Bivariate analysis showed no association between other demographic factors (age, marital status, education level, income generation, type of work and monthly income) and risk of HIV infection.

HIV- prevalence among participants with psychiatric conditions was 17.7% (12/68), versus 12.8% (12/94) among those with epilepsy (OR 1.46, 95% CI 0.55–3.84). No association was found between HIV status and the capacity to consent to the study, duration of symptoms or admittance into the central referral hospital.

### Risk Factors for HIV Acquisition

Of all patients, 129 (74.1%) did not report any physical health problems, 16 (9%) reported having undergone treatment for sexually transmitted infections, and two reported having been treated for tuberculosis. Ten participants reported use of alcohol, six binge drinking, four cigarette use and one cannabis use in the previous month.

The reported rate of sexual activity within the previous year was 61%. Of all sexually active participants, 15 (9%) had multiple (2 or more) partners. Among 91 participants with a stable relationship (married, engaged or living together), 11 (12%) had one or more other partners, 76 (89%) never or inconsistently used condoms with their partners and 46 (53%) reported not knowing the HIV status of their partner.

Among those with a stable relationship and additional sexual contact(s) 10 (91%) did not use condoms or used condoms inconsistently with their stable partner, and nine out of these 10 did not use condoms with additional sexual contact(s) either. Six (54%) reported not knowing the HIV status of their stable partner and seven (64%) that of other sexual partner(s).

Of 15 participants without a stable relationship with occasional sexual partners, ten reported never or inconsistently using a condom, seven of whom reported not knowing the HIV-status of their occasional sexual partners.

With regard to possible risk factors and HIV-infection, bivariate analysis showed that participants who reported physical health problems were more likely to be found HIV positive (OR 2.9, 95% CI 1.06–7.82). There were no associations between HIV-status and being sexually active, history of STI treatment, history of TB treatment, multiple sexual partners, condom use with spouse, condom use with non-spousal partner(s), alcohol use, binge drinking, cigarette and cannabis use and history of HIV testing. Also, no differences were found between those with epilepsy or psychiatric disorders with regard to the HIV risk factor variables.

Compared to female participants with a spouse, male participants with a spouse more often had one or more additional sexual partners. Alcohol use in the month prior to inclusion was higher among male than among female participants. No other differences between males and females were found with regard to possible risk factors for HIV acquisition (see table 2).

**Table 2:** Possible risk factors for HIV acquisition according to sex.

Variables	Males (N = 93)	Females (N = 81)	Chi squared test(two-sided p-value)
	n/N (%)		
**Diagnosis**			
Psychiatric disorder	43 (46)	32 (40)	p = 0,46
Epilepsy	50 (54)	49 (60)	p = 0.46
**Capacity to consent**	90 (97)	73 (90)	p = 0.13
**History of referral**	23 (25)	16 (20)	p = 0.55
**Ever tested for HIV pre- study (with documentation)**	28 (30)	37 (46)	p = 0.049
**Pre-study positive test results**	5 (5)	12 (15)	p = 0.067
**Sexual activity previous year**			
No sexual activity	34 (37)	33/80* (41)	p = 0.63
With spouse (+/− other partner(s))	53 (57)	38/80* (48)	p = 0.27
With only non-spousal partner(s)	6 (6)	9/80* (11)	p = 0.34
**Spouse + other sexual partner(s)**	11/53** (21)	0/38** (0)	p = 0.007
**2 or more sexual partners previous year**	12/92* (13)	3/79* (4)	p = 0.06
**Condom use spouse**			
Always	5/50* (10)	4/35* (11)	p = 0.88
Sometimes/never	45/50* (90)	31/35* (89)	p = 0.88
**Condom use non-spousal partner(s)**			
Always	2/17* (12)	3/9 (33)	p = 0.42
Sometimes/never	15/17* (88)	6/9 (67)	p = 0.42
**Knowledge HIV status spouse**			
No	24/50* (48)	22/36* (61)	p = 0.32
Yes, positive	0/50* (0)	2/36* (6)	p = 0.34
Yes, negative	26/50* (52)	12/36* (33)	p = 0.13
**Knowledge HIV status non-spousal partner(s)**			
No	11/17 (65)	5/9 (56)	p = 0.97
Yes, positive	0/17 (0)	0/9 (0)	
Yes, negative	6/17 (35)	4/9 (44)	p = 0.97
**Alcohol use previous month**	10 (11)	0 (0)	p = 0.0067

* denominator is total inclusions minus numbers unknown.

** denominator is total inclusions in subgroup minus numbers unknown.

### Comparison with the General Population

Those attending the mental health clinic population were less likely to be infected with HIV compared to the general population. Differences in HIV-prevalence between the clinic population (n = 24, 14.8%) and the general population (n = 271, 21.0%) [16] approached statistical significance (p = 0.08) (table 3).

**Table 3:** HIV prevalence general population of Thyolo District and clinic population.

	General population n/N (%)	Clinic population n/N (%)	Chi squared test (two-sided p-value)
**All**	271/1290 (21.0)	24/162 (14.8)	p = 0.08
**Men**	126/677 (18.6)	7/84 (8.3)	p = 0.03
**Women**	145/628 (23.1)	17/78 (21.8)	p = 0.91

Men in the clinic population were half as likely to be HIV-positive compared to men in the general population. The prevalence among women attending the clinic and women in the general population was comparable.

Of the clinic population, 97 persons (56%) completed standard 4 or more, compared to 243 persons (39%) in the sample of the general population (p<0.05); the clinic population had better access to electricity (n = 15 (9%) versus n = 250 (2%), p<0.05) [16].

Sexual activity in the past year was significantly lower in the clinic population compared to the general population (n = 106 (61%) versus n = 660 (84%), p<0.05), both for male participants (n = 59 (63%) versus n = 140 (83%), p<0,05), and female participants n = 47 (59%) versus 520 (84%), p<0.05). However, the percentage of men and women in the clinic population having multiple partners (n = 12 (13%) and n = 3 (4%)) were higher than the percentages of men (n = 15 (11%), p = 0.76) and women (n = 2 (0.4%), p<0.05) in the general population having multiple partners [16].

The uptake of HIV testing among the clinic population was high (n = 110 (63%)) compared to the 25% uptake of HIV testing among the adult population in Malawi [16]. No data about the uptake of HIV testing were available for Thyolo District.

## Discussion

This is the first published study of HIV among people with psychiatric disorders and epilepsy visiting an outpatient clinic in Sub-Saharan Africa. We found a trend towards a lower HIV-prevalence among this population, particularly men attending the clinic were significantly less likely to be infected than their male counterparts in the general population.

These findings differ from studies in high income countries that suggest that mental illness is a risk factor for developing HIV infection [5]-[8] and from previous studies from low-income countries with high HIV-prevalence, which showed that the prevalence among those with mental illness matched the general prevalence [4]; [10]. The latter studies were performed among inpatients. Outpatients generally have a better clinical condition than inpatients and may therefore show less high-risk sexual behaviour [15], suffer less often from social exclusion that may lead to exchange of sex for money or goods, and have less severe cognitive deficit that impairs judgement or the ability to negotiate safe sex [9].

We did not find an explanation for the finding that men in the study population had a lower HIV prevalence rate and women a similar prevalence rate compared to the general population: the rates of sexual activity of both men and women were comparable, and risk factors for HIV acquisition were either similar for men and women, or more prevalent among men. Both men and women were exposed to health promotion and other preventive measures during the monthly clinic visits.

Possible reasons for the relatively low number of HIV-positive men in our sample are that HIV-positive men in Malawi are less likely to seek health care [21] and have a higher mortality rate [22] compared to women. A combination of neuropsychiatric illness and HIV in a (remote) setting with poor health infrastructure increases mortality [23], and this effect may be more pronounced among men. Therefore, some men are likely to have died before they sought access to mental health or HIV care.

In addition, we found a lower rate of sexual activity among both men and women compared to the general population, which can be explained by stigmatization and sexual disinterest or dysfuntion due to neuropsychiatric symptoms and side effects of medication [6]. This low rate of sexual activity may have contributed to the lower HIV prevalence among men compared to the general population, but does not explain the similar prevalence among women. It could well be that some women with neuropsychiatric illness are more vulnerable to contract HIV compared to men for different reasons, such as being unable to negotiate safe sex. Vulnerability to sexual exploitation and high-risk sexual behaviour have been described for women in low income countries with severe mental illness and for women with epilepsy [24]; [25].

People with severe mental illness have higher all–cause mortality than those without [26]. It is possible that people with severe mental illness are not receiving recommended HIV-related healthcare, since the uptake of ART and cotrimoxazole prophylaxis among our HIV-positive patients were low. A high mortality rate would also explain the finding that patients from our sample with severe psychiatric symptoms (those who had been referred to the central referral hospital) had an even lower HIV prevalence compared to the sample prevalence (11.4% versus 14.8%), despite a high rate of sexual activity in this specific population (76%).

A number of possible study limitations merit discussion. Participants were recruited from a treatment site and results may not be generalizable to all people with mental illness. Second, a small number chose not to undergo HIV testing or were lost to follow up, so the sample may not be entirely representative. However, we estimate that these limitations had minimal effects on our findings: most people consented, the sample exceeded the calculated sample size and losses-to-follow up and opt-out rates were low.

The fact that the main researcher was one of the two doctors in the hospital and known by many of the patients attending the clinic, may have led to socially acceptable answers. Therefore, in reality, rates of high-risk sexual behaviour and substance abuse for instance, may be higher, and history of HIV testing lower than the rates reported. Discussing sexual risk behaviour with participants who lacked capacity to consent may have led to some inaccurate responses. However, we found that most participants in this relatively small group were able to discuss sexual behaviour.

The population attending the clinic was doing fairly well and symptoms generally appeared to be under control. The fact that the clinic functioned reasonably well, sustained an adequate drug supply, had a low staff turnover and provided weekly consultations is likely to have contributed. Furthermore, we found in the group of patients with psychiatric disorders very few dual diagnoses of psychiatric disorders and substance abuse (2.6%) compared to literature from high income countries [11] and low use of alcohol, cigarettes or cannabis. The educational and income levels of patients with psychiatric disorders indicate that the clinic population may be a sample of relatively stable patients who may not reflect the broader population of people living with psychiatric disorders in Malawi.

Our sample shows similar rates of sexual activity to studies from around the world [12]; [24]; [27]. Since the population data are not available, we could not compare the rate of high-risk sexual behaviours among sexually active patients in our sample to the general population. A comparison with other outpatient psychiatric populations shows a lower rate of multiple partners (9% versus 45% in the US [14] and 26.8% in Brazil [28]). However, the high rates of inconsistent condom use and lack of knowledge of the HIV-status of sexual partners indicate high levels of sexual risk behaviour.

Uptake of HIV testing was unexpectedly high. The general testing rate in Thyolo is unknown, and may be higher than the testing rate in Malawi due to a large HIV/AIDS project operating in the area. The high uptake of HTC may be related to high utilization of health services by the clinic population and a strong therapeutic alliance with care providers, similar to reports from developed countries [12]; [29]; [30]. In addition, the presence of well-organized HIV testing and health promotion messages, as well as the presence of HTC at the out-patient waiting area, may have contributed.

The finding from previous studies that patients with severe psychiatric symptoms were less likely to be tested [12]; [15]; [29]; [30] was not confirmed. We even found that the testing rate was higher among those with a history of severe psychiatric symptoms in need of referral to the central mental hospital. This may be due to HIV testing becoming a requirement before referral, and to increased knowledge about neuropsychiatric manifestations in HIV among clinicians. In addition, the increased availability of ART may have contributed positively.

However, mental health patients did not easily access treatment for their HIV disease. Many did not use co-trimoxazole prophylaxis or had not been assessed for ART eligibility. These findings may be caused by health workers’ reluctance to start ART or co-trimoxazole in mental health patients because of prejudice, fear of poor adherence, inadequate knowledge or high work pressure.

Our sample is a mixture of patients with mental illness and epilepsy. Little is known about HIV prevalence among people with epilepsy in low-income countries compared to patients with severe mental illness, or about social exclusion rates and cognitive deficit among this specific population. Those with epilepsy visiting the clinic were generally faring quite well, and had a low rate of monthly seizures. These patients were either able to visit the clinic by themselves or supported by caring guardians. This makes ‘social exclusion’ and ‘cognitive deficit’ probably minor factors in this specific population.

We found no specific patient-related factors that justify withholding HIV testing and care from patients with mental disorders. Almost all patients were found to have the capacity to consent to taking part in the study, or were accompanied by a capable caregiver. Most patients came in time for their drug refill, indicating good adherence.

In fact, the high rate of high-risk sexual behaviour among this population and the generally high HIV-prevalence in Thyolo District should encourage health staff to offer HTC to mental health patients. Our study suggests that patients with double diagnoses (and related stigma) of mental illness and HIV may miss out on services available within the health system.

## Conclusions

HIV-prevalence among patients attending a mental health clinic in rural Malawi was lower than in the general population, particularly due to a lower HIV-prevalence in males. The HIV testing rate was high, but uptake of preventive measures and HIV care low. Our findings indicate treatment gaps in: (1) male patients, who may delay seeking health care, and (2) all patients with neuropsychiatric illness, despite the fact that this clinic population was very well able to undergo testing and counselling for HIV. HIV prevention and care programmes should be available to patients with neuropsychiatric illness.

## References

[1] Murray CJ, Vos T, Lozano R, Naghavi M, Flaxman AD, et al. (2012) Disability-adjusted life years (DALYs) for 291 diseases and injuries in 21 regions, 1990–2010: a systematic analysis for the Global Burden of Disease Study 2010. Lancet, vol. 380, no.9859, pp2197–223. 10.1016/S0140-6736(12)61689-4 23245608

[2] Jacob KS, Sharan P, Mirza I, Garrido-Cumbrera M, Seedat S, et al (2007) Mental health systems in countries: where are we now? Lancet, vol. 370, no. 9592, 1061–1077. 17804052 10.1016/S0140-6736(07)61241-0

[3] Prince M, Patel V, Saxena S, Maj M, Maselko J, et al (2007) No health without mental health. Lancet, vol. 370, no. 9590, 859–877. 17804063 10.1016/S0140-6736(07)61238-0

[4] Collins PY, Berkman A, Mestry K, Pillai A (2009) HIV prevalence among men and women admitted to a South African public psychiatric hospital. AIDS Care, vol. 21, no. 7, 863–867. 20024743 10.1080/09540120802626188 PMC2800080

[5] Cournos F, McKinnon K (1997) HIV seroprevalence among people with severe mental illness in the United States: a critical review. Clinical Psychology Review, vol. 17, no. 3, 259–269. 9160176 10.1016/s0272-7358(97)00018-4

[6] Gottesman II, Groome CS (1997) HIV/AIDS risks as a consequence of schizophrenia. Schizophrenia Bulletin, vol. 23, no. 4, 675–684. 9366003 10.1093/schbul/23.4.675

[7] Rosenberg SD, Goodman LA, Osher FC, Swartz MS, Essock SM, et al (2001) Prevalence of HIV, hepatitis B, and hepatitis C in people with severe mental illness. American Journal of Public Health, vol. 91, no. 1, 31–37. 11189820 10.2105/ajph.91.1.31 PMC1446494

[8] Ayuso-Mateos JL, Montanes F, Lastra I, Pilcazo de la GJ, Ayuso-Gutierrez JL (1997) HIV infection in psychiatric patients: an unlinked anonymous study. British Journal of Psychiatry, 170, 181–185. 9093511 10.1192/bjp.170.2.181

[9] Collins PY, Holman AR, Freeman MC, Patel V (2006) What is the relevance of mental health to HIV/AIDS care and treatment programs in developing countries? A systematic review. AIDS, vol. 20, no. 12, 1571–1582. 16868437 10.1097/01.aids.0000238402.70379.d4 PMC2801555

[10] Acuda SW, Sebit MB (1996) Serostatus surveillance testing of HIV-I infection among Zimbabwean psychiatric inpatients, in Zimbabwe. Central African Journal of Medicine, vol. 42, no. 9, 254–257. 8997817

[11] Chander G, Himelhoch S, Moore RD (2006) Substance abuse and psychiatric disorders in HIV-positive patients: epidemiology and impact on antiretroviral therapy. Drugs, vol. 66, no. 6, 769–789. 16706551 10.2165/00003495-200666060-00004

[12] Meade CS, Sikkema KJ (2005) HIV risk behavior among adults with severe mental illness: a systematic review. Clinical Psychology Review, vol. 25, no. 4, 433–457. 15914265 10.1016/j.cpr.2005.02.001

[13] Campos MD, Podus D, Anglin MD, Warda U (2008) Mental health need and substance abuse problem risk: acculturation among Latinas as a protective factor among CalWORKs applicants and recipients. Journal of Ethnicity in Substance Abuse, vol. 7, no. 3, 268–291. 19042810 10.1080/15332640802313262

[14] Meade CS, Sikkema KJ (2007) Psychiatric and psychosocial correlates of sexual risk behavior among adults with severe mental illness. Community Mental Health Journal, vol. 43, no. 2, 153–169. 17143728 10.1007/s10597-006-9071-6

[15] Senn TE, Carey MP (2009) HIV testing among individuals with a severe mental illness: review, suggestions for research, and clinical implications. Psychol.Med., vol. 39, no. 3, 355–363. 18606051 10.1017/S0033291708003930 PMC2640447

[16] National Statistical Office (2005) Malawi Demographic and Health Survey 2004. Government of Malawi, Lilongwe

[17] Bemelmans M, Van den Akker T, Ford N, Philips M, Zachariah R, et al (2010) Providing universal access to antiretroviral therapy in Thyolo, Malawi through task shifting and decentralization of HIV/AIDS care. Tropical medicine & international health, vol. 15, no. 12, 1413–1420. 20958897 10.1111/j.1365-3156.2010.02649.x

[18] Government of Malawi, Ministry of Health (2008) Guidelines for the use of antiretroviral therapy in Malawi, 3rd edition. Lilongwe.

[19] Government of Malawi, Ministry of Health (2009). Guidelines for HIV Testing and Counselling, 3rd edition. Lilongwe.

[20] Sullivan KM, Dean A, Soe MM (2009) OpenEpi: a web-based epidemiologic and statistical calculator for public health. Public Health Report, vol. 124, no. 3, 471–474. 10.1177/003335490912400320 PMC2663701 19445426

[21] Parrot FR, Mwafulira C, Ngwira B, Nhwazi S, Floyd S, et al (2011) Combining qualitative and quantitative evidence to determine factors leading to late presentation for antiretroviral therapy in Malawi. PloS One, vol. 6, no. 11, e27917. 22114727 10.1371/journal.pone.0027917 PMC3218069

[22] Weigel R, Estill J, Egger M, Harries AD, Makombe S, et al (2012) Mortality and loss to follow-up in the first year of ART: Malawi national ART programme. AIDS, vol. 26, no.3, 365–73. 22095194 10.1097/QAD.0b013e32834ed814 PMC3811026

[23] World Health Organization (2011b) World Health Statistics Geneva, WHO.

[24] Chandra PS, Carey MP, Carey KB, Prasada Rao PS, Jairam KR, et al (2003) HIV risk behaviour among psychiatric inpatients: results from a hospital-wide screening study in southern India. International Journal of STDs and AIDS, vol. 14, no. 8, 532–538. 10.1258/095646203767869147 PMC2424168 12935383

[25] Birbeck G, Chomba E, Atadzhanov M, Mbewe E, Haworth A (2007) The social and economic impact of epilepsy in Zambia: a cross-sectional study. Lancet Neurology, vol. 6, no. 1, 39–44. 17166800 10.1016/S1474-4422(06)70629-9 PMC2938018

[26] Colton CW, Manderscheid RW (2006) Congruencies in increased mortality rates, years of potential life lost, and causes of death among public mental health clients in eight states. Prev Chronic Dis 3: A42. 16539783 PMC1563985

[27] Tucker JS, Kanouse DE, Miu A, Koegel P, Sullivan G (2003) HIV risk behaviors and their correlates among HIV-positive adults with serious mental illness. AIDS Behavior, vol. 7, no. 1, 29–40. 14534388 10.1023/a:1022557222690

[28] Wainberg ML, McKinnon K, Elkington KS, Mattos PE, Gruber MC, et al (2008) HIV risk behaviors among outpatients with severe mental illness in Rio de Janeiro, Brazil. World Psychiatry, vol. 7, no. 3, 166–172. 18836542 10.1002/j.2051-5545.2008.tb00190.x PMC2559926

[29] Desai MM, Rosenheck RA (2004) HIV testing and receipt of test results among homeless persons with serious mental illness. American Journal of Psychiatry, vol. 161, no. 12, 2287–2294. 15569902 10.1176/appi.ajp.161.12.2287

[30] Desai MM, Rosenheck RA, Desai RA (2007) Prevalence and correlates of human immunodeficiency virus testing and posttest counseling among outpatients with serious mental illness. The Journal of nervous and mental disease, vol. 195, no. 9, 776–780. 17984780 10.1097/NMD.0b013e31814514ad

